# Association of Proton Pump Inhibitor Therapy with Hepatic Encephalopathy in Hepatitis B Virus-related Acute-on-Chronic Liver Failure

**DOI:** 10.5812/hepatmon.16258

**Published:** 2014-04-07

**Authors:** Zhao-Ni Lin, Yong-Qing Zuo, Peng Hu

**Affiliations:** 1 Department of Infectious Diseases, Institute for Viral Hepatitis, Key Laboratory of Molecular Biology for Infectious Diseases, Ministry of Education, Second Affiliated Hospital of Chongqing Medical University, Chongqing, China

**Keywords:** *Hepatic Encephalopathy*, *Proton Pump Inhibitors*, *Hepatitis B Virus*

## Abstract

**Background:**

Hepatic encephalopathy (HE) is an important neuropsychiatry complication of acute-on-chronic liver failure (ACLF). PPI therapy may increase the intestinal bacterial overgrowth and infections.

**Objectives:**

The aim of this study was to assess whether PPI use in ACLF is associated with HE.

**Patients and Methods:**

A retrospective case-control study was performed. Fifty five admitted patients with hepatitis B virus (HBV)-related ACLF complicated by Stage II-IV HE developed after admission between January 2008 and December 2012 were matched (by sex, age, and MELD score) with comparable HBV-related ACLF patients (n = 110) who did not develop this complication during hospitalization. We excluded combined HE upon admission and other neurological disorders in patients with ACLF. Univariate and multivariate analyses of 30 variables (laboratory examination, predisposition, treatment, etc.) before the occurrence of HE were carried out to identify the factors predictive of HE.

**Results:**

In univariate analysis, patients with HE in ACLF had a significantly higher rate of PPI use (89.1%) compared with non-HE (63.6%, P = 0.001). In addition, clinical and standard laboratory variables were significantly different between the two groups regarding the infection rate, hyponatremia, alpha-fetoprotein (AFP), Arginine Hydrochloride use and Lactulose use. Logistic regression analysis was used to examine the combined effects of the variables with HE as the outcome. HE in ACLF was associated with hyponatremia (odds ratio (OR) = 6. 318, 95% confidence interval (CI) = 2. 803-14.241; P = 0. 000), PPI use was independently associated with HE (OR = 4. 392, CI = 1. 604-12.031; P = 0. 004), and lactulose use was protective (OR = 0. 294, CI = 0. 136-0.675; P = 0. 003).

**Conclusions:**

The occurrence of HE is associated with hyponatremia and PPI use in patients with ACLF.

## 1. Background

Acute-on-chronic liver failure (ACLF) is difficult to treat and carries a high risk of short-term mortality (1, 2), and may result in life-threatening complications such as hepatic encephalopathy (HE), infection, bleeding and hepatorenal syndrome (HRS). HE is a complex and progressive neuropsychiatric syndrome, which is one of the common manifestations of ACLF in the absence of other neurological disorders. It is characterized by changes in mental state, including a wide range of neuropsychiatric symptoms ranging from minor signs of altered brain function to deep coma. Gut-derived nitrogenous substances are universally acknowledged to play a major role in the pathogenesis of HE (3, 4). ACLF usually results following a precipitating event in the context of established cirrhosis. Patients with liver cirrhosis have been found to have substantial derangements in the gut microecology, with significant fecal overgrowth of potentially pathogenic Escherichia coli and Staphylococcal species. Small intestinal bacterial overgrowth (SIBO) in cirrhotic patients is common and associated with systemic endotoxemia, even in the absence of overt infection (5). In addition, abnormal intestinal motility may play an important role in increasing the growth of pathogenic bacteria and the absorption of gut toxins. These may increase the intestinal absorption of ammonia that leads to an increase in the occurrence of HE. The primary treatment of HE is reducing or eliminating the increased neurotoxic ammonia levels based on the identification and treatment of the precipitating factors. Lactulose (6) and non-absorbable antibiotics (7), remain the mainstay treatment for HE.

Proton pump inhibitors (PPIs) are commonly prescribed in cirrhosis to prevent the portal hypertension bleeding, which are known to have an excellent safety proﬁle. However, in a minority of patients, PPIs may be prescribed without clear indications or because of their propensity to develop upper gastrointestinal symptoms. PPIs, which act by reducing acid secretion, could increase the risk of gastrointestinal (GI) infections by raising the pH of the stomach and making it more prone to colonization by various pathogenic bacteria. The PPIs can disrupt the gut ecology too, they change the bacterial growth, including abnormal bacterial counts and overt SIBO (8). Moreover, gastric acid influences not only the upper gut flora, but also lower intestinal microflora. The number of bacteria in small and large bowel increases as a result of gastric hypochlorhydric conditions (9). Previous case control studies have found an increased risk of GI infections in patients taking PPIs (10). Some studies have reported that PPI therapy is associated with spontaneous bacterial peritonitis (SBP) in patients with advanced cirrhosis (11, 12). Increased ammonia-producing enteric bacteria in patients is shown to be a risk factor for HE (13). Besides, considering that patients with ACLF have a high prevalence of gastrointestinal symptoms, PPIs may increase absorption of gut-derived nitrogenous substances because of its enhancing effect on retarding gastrointestinal motility (14), delaying gastric emptying rate and decreasing gastric mucus viscosity. HE can occur either due to liver failure or due to one or more precipitating factors in a cirrhotic patient. However, these neuropsychiatric manifestations are potentially reversible, if the relevant prognostic factors for HE could be clarified, appropriate measures could be adopted to reduce the associated mortality. Numerous studies have been published concerning the prognostic factors in ACLF (15), but there is little information regarding the factors predictive of development of HE. Nevertheless, it has been recently hypothesized that PPI therapy may increase the intestinal bacterial overgrowth (11, 16), which produces more toxic substances in colon.

## 2. Objectives

The goal of the current study was to determine whether PPI use in patients with ACLF is associated with subsequent development of HE, and to compare it with other simultaneously measured clinical variables. 

## 3. Patients and Methods

### 3.1. Patients and Study Design

The medical records from all ACLF patients consecutively admitted to hospital at the second affiliated hospital of Chongqing Medical University between January 2008 and December 2012 were retrospectively reviewed. At the beginning of the study, we estimated the optimal sample size of this case control study by applying the frequency matching-design related formula. In order to improve the study efficiency and control for confounding factors, the case and control subjects were matched by sex, age, and MELD score (frequency matching). In China, as a result of the high prevalence of hepatitis B virus (HBV), chronic HBV infection is the most common cause of liver failure (17, 18). Inclusion criteria included: 1) Hepatitis B virus-related acute-on-chronic liver failure 2) Patients who had their first episode of Grade II-IV HE in the hospital for at least six days were included in the study. Exclusion criteria included: combined HE patients on admission or patients who had their first episode of HE before the sixth day after the admission (because the goal of this study was to evaluate the independent association between PPI use and HE in patients with ACLF). PPIs should be used more than 5 days to cause stable lasting acid suppression (gastric pH > 4) (19-21). Also patients who had their first episode of HE before the sixth day after the admission may not be a good candidate for studying the association between PPI and HE); concurrent viral infections (e.g. HAV, HCV, HDV, HEV, human immunodeficiency virus, cytomegalovirus, Epstein-Barr virus); liver failure due to other causes (including autoimmune, alcohol- or drug-related diseases); malignant tumors; TIPS (the proportion of these patients are few); and patients with other severe systematic or mental diseases. The study was approved by the Institutional Review Boards (IRB) for Human Subject Review at The Second Affiliated Hospital of Chongqing Medical University and all aspects of the study comply with the declaration of Helsinki. IRB committee approved that no informed consent was required because the data were going to be analyzed anonymously.

### 3.2. Related Definitions

Acute-on-chronic hepatitis B liver failure, as deﬁned by the APASL working party, is acute hepatic insult manifesting as jaundice (serum bilirubin > 5 mg/dL) and coagulopathy (international normalized ratio [INR] > 1.5 or prothrombin activity < 40%), complicated within 4 weeks by ascites and/or encephalopathy in a patient previously diagnosed or undiagnosed with chronic liver disease (22). The severity of HE was graded as: grade 1: Euphoria or depression, mildly disturbed sleep-awake cycle, hypersomnia, insomnia; grade 2: Inappropriate behavior, disorientation, apathy, mood swings; grade 3: Very sleepy but arousable, unresponsive to verbal commands, markedly confused, combative and hyper-reflexic, and grade 4: Unconscious, coma (13). Hyponatraemia was deﬁned as serum sodium concentration < 135 (mmol/L) (should have developed at least one episode of hyponatraemia before the occurrence of HE). PPI use definition: patients using any PPI intravenously for at least six days before the occurrence of HE at the admission time were counted as PPI users, others were considered to be non-users. Hospitalized patients of ACLF had serious medical condition, and all received PPI intravenously in the study.

### 3.3. Recorded Parameters

Clinical and laboratory information was collected at the time of admission. Information included age, sex, MELD score; major precipitating factor before the occurrence of HE during the stay in hospital, such as: infection, upper gastrointestinal bleeding, electrolyte disorder, PPI use, lactulose use, drugs to prevent hepatic coma, use of intestinal probiotic preparations and oral antibiotics; laboratory data including albumin, aminotransferase, r-GGT, total bilirubin, direct bilirubin, and creatinine levels, urea nitrogen, and the international normalized ratio (INR) of the prothrombin time, prothrombin activity and serological tests for hepatitis B surface antigen (HBsAg), hepatitis Be antigen (HBeAg), and anti-HBe which were measured by commercially available enzyme-linked immunoassays. HBV DNA estimation was done with the real-time polymerase chain reaction (PCR) method (lower limit of detection = 50 IU/mL, Roche Taqman assay). Of the 30 variables included in the univariate analysis, we included the variables with P < 0.1 which were clinically a common cause of HE into the Logistic regression. In the multivariate analysis, 14 variables were included in a stepwise regression.

### 3.4. Statistical Analysis

Statistical analyses were performed using SPSS software version 17.0. Student’s t-tests or the non-parametric Wilcoxon rank sum tests were used to compare Patients’ clinical and biochemical indices, and Fisher’s exact tests or Pearson’s chi-square tests were used for the categorical variables. Continuous variables are expressed as mean ± standard deviation (SD); categorical variables are expressed as frequency and percentages. A P value of < 0.05 was considered statistically significant. Multivariate analysis was performed by logistic regression analysis using stepwise selection (LR), and the statistical cut points giving the best possible results for sensitivity and speciﬁcity of the statistical models were determined. Estimations of risks were made using 95% conﬁdence intervals and their associated p-value. Survival curves were derived by the Kaplan-Meier method.

## 4. Results

**Figure 1.**
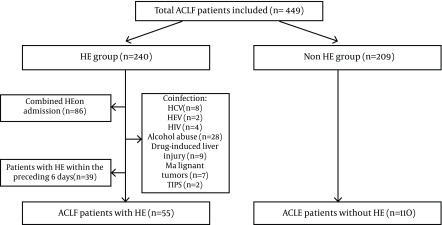
Flow Diagram of the Study Disposition

Charts of 449 patients with ACLF were reviewed. Fifty five consecutive hospitalized patients with HBV-ACLF who met the inclusion and exclusion criteria were enrolled in this study. In the same admission time period, 110 comparable HBV-ACLF patients without HE during hospitalization were selected as the control group, using frequency matching (by sex, age and MELD score) (Figure 1). There were no significant differences with regards to sex (males 83.6% vs. 75.5%, P = 0. 23), age (46 (37-55) vs. 43 (36-48), P = 0. 068), and MELD score (25.3 ± 5 vs. 24.6 ± 5.9, P = 0. 515) between the two groups. Interestingly, patients with HE in HBV-ACLF had a significantly higher rate of PPI use (89.1%) compared with non-HE patients (63.6%, P = 0.001) (Table 1). In addition, the survival rates of patients in the non-HE group (68.2%) were higher than those of the patients in the HE group (20%) (P = 0.000).

The baseline clinical and biochemical characteristics of HE and non-HE groups are shown in Table 1. The laboratory data and virological characteristics were assessed at admission. No significant differences were observed between HE and non-HE patients with cirrhosis (50.9% vs. 51.8%, P = 0.912) and ascites (74.5% vs. 60.9%, P =0.082). The level of AFP was significantly lower in patients with HE (111.6 (13-65.6)) compared with those without HE (172.7 (13-207), P = 0. 005). Otherwise, the laboratory picture was similar between the two groups, such as: RBC, HB, WBC, PLT, ALB, ALT, AST, TB, DB, GGT, Creatinine levels, Urea nitrogen, PTA%, HBV-DNA load, HBeAg status (P > 0.05). We investigated the precipitating events associated with the development of HE. Among patients with HE, 44 of 55 patients (80.0%) had infection which was significantly higher than 68 of the 110 non-HE patients (61.8%, P =0.018). Furthermore, 29 out of 55 patients with HE (52.7%) had hyponatraemia, compared with only 18 of the 110 patients (16.4%) in HBV-ACLF patients without HE, this difference was highly significant (P = 0.000). There was no difference between two groups regarding the upper gastrointestinal bleeding before the occurrence of HE during hospitalization period (9.1% vs.5.5%, P = 0. 377). We also investigated the use of medications before the occurrence of HE after admission. Among patients with HE, the proportion of patients taking PPI was 89.1%, compared with only 68.2% in ACLF patients without HE. This difference was highly significant (P = 0. 001). Indications for PPI prescription also were similar in the two groups. The proportion of patients with HE taking oral lactulose before the occurrence of HE was 32.7%, compared with 50.9% in patients without HE, the difference was significant (P = 0. 027). Two groups were significantly different regarding the use of arginine Hydrochloride (21.8% vs. 10.0%, P =0.039), but there were no significant differences between the two groups regarding use of oral antibiotics, drugs to prevent hepatic coma (3AA, LOLA) and use of intestinal probiotic preparations (P > 0.05) (Table 1). Logistic regression was used to examine the combined effects of the variables with HE as the outcome. HE in HBV-ACLF was associated with hyponatremia (odds ratio (OR) = 6. 318, 95%, Confidence interval (CI) = 2, 803-14.241; P = 0. 000), PPI use was independently associated with HE (OR = 4. 392, CI = 1. 604–12.031; P = 0. 004), and lactulose use had a protective effect (OR = 0. 294, CI = 0. 136–0.675; P = 0. 003) (Table 2). 

**Table 1.** Comparison of Clinical, Biochemical and Virological Characteristics of Patients in Two Groups Using Univariate Analysis ^a^, ^b^

	HE (n=55)	Non HE (n=110)	P Value
**Age, y**	46 (37-55)	43 (36-48)	0.068
**Gender**			0.230
Men	46	83	
Women	9	27	
**MELD Score**	25.3 ± 5	24.6 ± 5.9	0.515
**Upper gastrointestinal bleeding, %**	5 (9.1)	6 (5.5)	0.377
**Infection, %**	44 (80.0)	68 (61.8)	0.018
**Hypokalemia, %**	17 (30.9)	20 (18.8)	0.065
**Hyponatraemia, %**	29 (52.7)	18 (16.4)	0.000
**Cirrhosis, %**	28 (50.9)	57 (51.8)	0.912
**Ascites, %**	41 (74.5)	67 (60.9)	0.082
**RBC, 10**^**12**^**/L**	3.7 ± 0.9	3.8 ± 0.8	0.214
**HB, g/L**	117.6 ± 24.7	119.0 ± 21.5	0.708
**WBC, 10**^**9**^**/L**	6.2 ± 3.1	6.2 ± 2.8	0.994
**PLT, 10**^**9**^**/L**	80.9 ± 45	85.9 ± 47	0.516
**Total protein, g/L**	63.5 ± 8.4	65.0 ± 8.0	0.141
**ALB, g/L**	30.8 ± 6.3	31.1 ± 4.9	0.716
**ALT, IU/L**	548.5 (68-959)	614 (93-1015)	0.483
**AST, IU/L**	598.1 (107-1065)	518.3 (119-763)	0.737
**TB, umol/L**	285.8 (175-282)	275.0 (163-363)	0.605
**DB, umol/L**	201.5 (126-281)	194.0 (112-261)	0.609
**GGT, umol/L**	88.7 (42-131)	92.2 (48.5-116.5)	0.473
**Serum creatinine, umol/L**	73 (58-88)	75 (57-84)	0.54
**Urea nitrogen, umol/L**	4.5 (3-5.4)	5.0 (3.0-5.3)	0.434
**PTA, %**	29 ± 7	31 ± 7	0.09
**AFP, ug/L**	111.6 (13-65.6)	172.7 (13-207)	0.005
**HBV-DNA, Log**_**10 **_**IU/L**	5.8 (4.2-7.7)	5.4 (3.4-6.8)	0.262
**HBe Ag-positive, %**	28 (50.9)	53 (48.2)	0.741
**oral antibiotics, %**	7 (13.7)	9 (8.2)	0.352
**Probiotic preparations, %**	15 (27.3)	34 (30.9)	0.630
**Lactulose use, %**	18 (32.7)	56 (50.9)	0.027
**Branched chain amino acids, %**	16 (29.1)	19 (17.3)	0.080
**L-ornithine-L-aspartate, %**	13 (23.6)	29 (26.4)	0.705
**Arginine hydrochloride, %**	12 (21.8)	11 (10)	0.039
**PPI use, %**	49 (89.1)	70 (63.6)	0.001

^a^ Abbreviations: AFP, alpha-fetoprotein; ALT, alanine aminotransferase; AST, aspartate aminotransferase; DB, direct bilirubin; GGT, glutamyltransferase; HB, hemoglobin; MELD, model for end-stage liver disease; PPI, proton pump inhibitor; PTA, prothrombin activity; RBC, Red blood cell count; TB, total bilirubin;WBC, White blood cell count.

^b^ Data are presented in Mean ± SD or No. (%).

**Table 2.** Logistic Regression (by Forward LR) Was Used to Examine the Combined Effects of the Variables (HE was designated as the Outcome)

	Odds Ratio	95% Confidence Interval	P Value
**Hyponatraemia**	6.318	2.803-14.241	0.000
**Lactulose use**	0.303	0.136-0.675	0.003
**PPI use**	4.392	1.604-12.031	0.004

**Figure 2.**
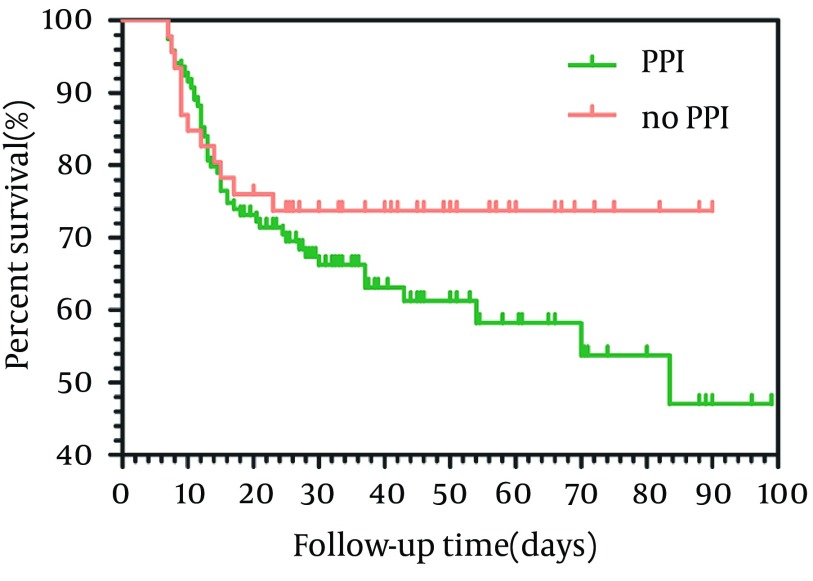
Kaplan–Meier Survival of the Two Groups of Patients With and Without Indication of PPI Use Who Were Followed for 90 Days

In our study, 49 patients were prescribed PPIs before HE, but there were only 15 patients who had clear indication for PPIs use; in other patients there were no clear indications for PPI prescription, for example, PPIs were prescribed just for some gastrointestinal symptoms, such as abdominal pain and dyspepsia (Table 3). In addition, we assessed the mortality rates of patients with and without clear indication of PPI. The 3-months mortality of ACLF patients using PPIs reached 37.8% (45.119), compared with only 26.1% (12.46) in ACLF patients not taking PPIs. As shown in Figure 2, there was no signiﬁcant difference in the overall survival rate between the two groups (95% Confidence interval = 60. 6-73.9, P = 0. 212).

**Table 3.** Detailed Regimens of Proton Pump Inhibitor ^a^, ^b^

	HE Group PPI Users (n = 49)	Non-HE Group PPI Users (n = 70)	P Value
**Indication for PPI Therapy**			
GERD	1 (2)	2 (2.9)	1.000
Peptic ulcer	2 (4.1)	6 (8.6)	0.555
Prophylactic use of PPI for the portal hypertension bleeding	8 (16.3)	18 (25.7)	0.223
Upper gastrointestinal bleeding	4 (8.2)	4 (5.7)	0.600
No documented indication	34 (69.4)	40 (57.1)	0.175

^a^ Abbreviations: GERD, Gastroesophageal reflux disease; PPI, proton pump inhibitor.

^b^ Data are presented in No. (%).

## 5. Discussion

In the current retrospective case – control study, 165 of HBV-ACLF patients were analyzed with the aim of assessing factors associated with the development of HE. Our data supported an association between PPI use and developing HE in patients with ACLF. PPI prophylaxis for gastrointestinal bleeding and variceal or portal hypertension are a common clinical practice. In addition, liver failure patients often have severe gastrointestinal symptoms such as: reflux, nausea, vomiting, and abdominal pain. So, PPIs which are commonly used to suppress the secretion of stomach acid are used to alleviate symptoms of the digestive tract. The adverse consequences of PPI use have been increasingly reported, though it has an overall good safety profile. Gastric acid has the capability of killing exogenous acid sensitive bacteria introduced into the stomach, usually within 15 minutes at the pH < 4. Any factors which increase the gastric pH above four may cause a state of hypochlorhydria and potentially increases the susceptibility to various microbes, including enteropathogenic forms with the potential to colonize, invade or inﬂame the intestine, allowing at least 50% of the ingested bacteria to survive the gastric trap (23). Animal model studies have shown that using gastric acid inhibitors can cause bacterial translocation in a way that enteric microbes will be able to escape the local gut defenses and epithelial barrier (24). It should be noted that the studies about the effect of PPIs on bacterial translocation were limited to animal studies. Recently published studies have suggested that PPIs could increase the risk of SBP in patients with cirrhosis (11, 12, 16, 25, 26). The use of PPIs increases the gastric pH, bacterial translocation, facilitates the growth of the gut microflora, and alters various immunomodulatory and anti-inflammatory effects (12, 27). Therefore, the attenuation of immune system provides a plausible mechanism to explain why patients consuming PPIs might be at increased risk of SBP. For Salmonella and C. jejuni strains, the relatively few published studies report a signiﬁcant association of enteric infections with PPI use (10, 28-31), and this situation was similar to anti-histamine receptor antagonist use (32). Besides, it was reported that bacterial pneumonias (33-35) and hip fracture (36, 37) might be more common in patients on PPIs.

However, there were few studies concerning the association of PPI therapy with HE in patients with ACLF. Increased gastric PH, as a consequence of PPIs, small intestinal bacterial overgrowth (SIBO) (8), and abnormal intestinal motility (14) may play an important role in increasing the growth of pathogenic bacteria and increased absorption of gut toxins in the portal system, which may lead to an increased incidence of HE especially in ACLF. In our study, logistic regression analysis showed that PPI use was independently associated with HE (OR = 4.392, CI = 1.604–12.031; P = 0.004), which was attributed to increased ammonia as a key factor in the pathogenesis of HE. Most therapies for HE focus on treating episodes as they occur and are directed at reducing the nitrogenous load in the gut, an approach that was consistent with the hypothesis that this disorder results from the systemic accumulation of gut-derived neurotoxins, especially ammonia, in patients with impaired liver function . It is also previously shown that oral antibiotics and lactulose can prevent HE (7, 38). In addition, PPIs suppress gastric acid secretion and decrease the severity of symptoms of indigestion compared with patients without PPI (39). So, PPIs should be more cautiously used in patients with ACLF. PPIs have provided benefits in the management of gastrointestinal diseases, including gastroesophageal reflux disease and peptic ulcer disease, but unfortunately, the unnecessary prescription of PPIs has become an important problem, which increases economic costs in daily clinical practice (40). Meanwhile, a clinical trial demonstrated that prophylactic use of PPIs did not improve the portal hypertension-related bleeding in patients with cirrhosis (41). Besides, patients with ACLF have a high prevalence of gastrointestinal symptoms, and PPIs may alter gastric emptying, thus aggravating the digestive tract symptoms. A recent retrospective study by Bajaj et al. (12) showed that 47% of cirrhotic patients receiving PPI had no documented indication for PPI treatment. In our study, 69.4 % (n = 34) of these patients had inadequate indications for PPI treatment in the HE group, as well as 57.1% of patients (n = 40) in non-HE gtroup. This finding was consistent with previous reports (39). Therefore, it might be beneficial that a valid indication for PPI use would be applied in daily clinical practice. We must point out the limitations of our study, the first of which is that this was a retrospective analysis of HE. The diagnosis of HE was based on the clinical criteria. Thus, it was difficult to assess patients who had a minimal degree of HE or HE with grade less than II, because the related symptoms can be easily ignored. So these types of patients failed to enter into our study. In addition, patients' use of PPIs before the admission was not specified in the medical records. Another limitation of our study was that the collection of the information related to plasma ammonia levels was not complete. It was better if ammonia levels at the time of admission and the occurrence of HE were recorded, and entered into the univariate and multivariate analyses. This might have made our results more convincing. In conclusion, the occurrence of HE was related to hyponatremia, and PPI use in patients with HBV-ACLF. Lactulose was effective in the prevention of HE in patients. Prospective studies are needed to determine whether PPI avoidance can reduce the incidence of HE and improve patients' outcomes.
